# Exploring the timing of medical student research internships: before or after clerkships?

**DOI:** 10.1186/s12909-018-1367-z

**Published:** 2018-11-12

**Authors:** Inge J. van Wijk, Hester E. M. Daelmans, Anouk Wouters, Gerda Croiset, Rashmi A. Kusurkar

**Affiliations:** 1 Department of Pediatrics, VU University Medical Center, PO BOX 7057, 1007 MB Amsterdam, the Netherlands; 2 VUmc School of Medical Sciences, VU University Medical Center, Amsterdam, the Netherlands; 3 LEARN! Research institute for learning and education. Faculty of Psychology and Education, VU University, Amsterdam, the Netherlands; 4 University Medical Center Groningen, Groningen, the Netherlands

**Keywords:** Research internship, Curriculum, Position, Self determination theory, Motivation

## Abstract

**Background:**

The objective of this study was to determine the optimal positioning of the research internship, either before clinical clerkships, at the beginning of the medical Master’s programme, or at the end.

**Methods:**

A mixed methods study was carried out. We compared characteristics such as duration, location and grades for internships performed and students’ motives for choosing to perform their research internship *before* or *after* clinical clerkships. We analysed students’ answers to open-ended questions about the reasons for their choices, using the Self-Determination Theory of motivation.

**Results:**

Students performing their research internship *before* clinical clerkships (*n* = 338) opted more often for an extended internship (OR = 3.16, 95% CI = 2.32–4.31) and an international location (OR = 2.22, 95% CI = 1.46–3.36) compared to those performing their research internships *after* clinical clerkships (*n* = 459). Neither the internship grades nor the number of international publications differed significantly between the two groups. Most of the students’ motives (102 participants) were classified as extrinsic motivation for research. Students performing research *before* clinical clerkships more often showed intrinsic motivation for research, students performing research *after* clinical clerkships were mainly motivated by their career choice.

**Conclusion:**

To accommodate both groups of students, offering research internships *before* and *after* clinical clerkships, is recommended.

## Background

Medical students need to acquire research skills and learn critical inquiry, reasoning and appraisal in order to make clinical decisions using research outcomes and to be able to translate research findings into daily patient care. Therefore, training medical students in performing and understanding clinical and translational research is a valuable part of the medical curriculum. In most medical curricula with a mandatory research internship, this training is placed at the end of the curriculum. Yet, the optimal timing of such training in the medical curriculum is unknown. The present study explores two different positions of a research internship in a medical curriculum, to determine the optimal timing to perform research during the medical studies.

Research training might be placed beyond the core curriculum as an elective, a summer course or in an intercalated year [1–4]. Although there is no consistent way in which medical students are engaged in research, many medical schools now have integrated some research training within their formal curriculum [5–8]. Feldman [9] states that the accreditation standards established by the Association of American Medical Colleges [10] specifically require that a medical school includes an educational programme in translational research in its curriculum. Likewise, Lemon and co-workers [11] and Abu Zaid [12] make a plea for a research training in all medical curricula.

Students who were exposed to research early in their curriculum (in preclinical education) expressed a significantly higher interest in performing research in the future [13, 14]. In parallel, Feldman argues that a research experience should be introduced to students as early as possible in the curriculum to enable students to pursue a research project throughout their programme [9]. However, a mixed methods study performed by Rosenkranz and co-workers reported higher future intentions pursuing research post-graduation, in students who were more advanced in their clinical programme [15]. Thus, it remains unclear what the best choice is: an early research experience or a research internship at the end of the medical studies?

In the Netherlands, a compulsory research internship is part of all medical curricula. A period of four to 6 months is allocated for students to learn how to perform scientific clinical and translational research by participating in research in a full time and hands-on manner. Students are free to choose to carry out e.g. laboratory (bench) research, clinical research, qualitative research, literature reviews etc. Students learn to define a research problem, search and appraise literature, formulate research questions and write a research proposal. They subsequently collect, analyse and interpret data, write a research report within 3 months after finishing the project, and present and discuss the results orally [16, 17]. Some students publish the results of their internship in an international scientific journal [16].

**Fig. 1:**
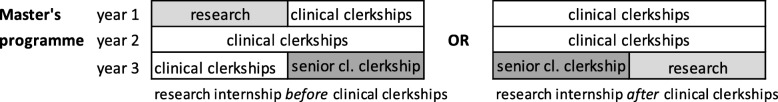
Position of the research internship in the medical Master’s curriculum of VU University Medical Center

In Dutch medical schools the medical studies consist of a three-years Bachelor’s and a three-years Master’s programme. In these Master’s programmes, the research internship is most frequently performed after students have completed all clinical clerkships, i.e. towards the end of their medical studies. At the VU University Medical Center in Amsterdam we offer students a choice in their Master’s programme: they can either perform their research training in the first year or in the third year of their Master’s programme (see Fig. 1). In addition, students are free to choose the location, department, research project (as long as projects meet all quality standards) and duration of the research internship (16 or 24 weeks).

As students learn about research methods, statistics, literature searches and acquire scientific writing skills during their Bachelor’s programme, they are well-prepared to start the research internship at the start of the Master’s programme.

Although in many medical curricula, the research internship is placed at the end of the programme, there is internationally no consensus about the optimal timing of the research internship.

The current study made use of the unique situation that in one curriculum students are able to perform their mandatory research internships either *before* or *after* their clinical clerkships. As a proxy for effective learning we used the grades of the internships and numbers of publications derived from the research performed. We compared these quantitative characteristics between research internships conducted *before* and *after* clinical clerkships. Moreover we aimed to understand how students make their choice for one of the two options. Reasons given by students were analysed in order to obtain an insight into students’ motives and expectations and to identify potential differences between these groups of students, thereby addressing the question on optimal timing of the internship.

### Theoretical framework

The framework of the Self-Determination Theory (SDT) [18] was used to explore student motivation for research and the goals in choosing to do the research internship *before* or *after* their clinical clerkships. SDT classifies the types of motivation for engaging in an activity as intrinsic regulation, identified regulation, introjected regulation and extrinsic regulation. Intrinsic regulation is characterized by inherent interest or personal endorsement of an activity. For example: “I perform research because I am curious and like to explore unsolved medical problems”. Identified regulation involves a conscious acceptance of the behaviour as being important to achieve a certain goal. For example: “I perform research because I think this will make me a better doctor”. Introjected regulation involves the internalization of external pressure, a sense of obligation is felt and the source is guilt, worry or shame. For example: “I perform research because as a future physician, I am supposed to”. External regulation involves activities being done out of external pressure or to obtain rewards. For example: “I perform research because it might help me to obtain a position as a resident”.

## Methods

### Study design and sample

The study is a mixed methods study using quantitative characteristics and outcomes of research internships, and a qualitative inquiry of students’ reasons for making their choice to carry out their research internship *before* or *after* their clinical clerkships.

All students who had finished their research internship in the Master of Medicine at the VU University Medical Center between September 2013 and September 2016 were included in the quantitative part of the study. However, for the analysis of publications, internship data from September 2012 to September 2014 were used.

From February 2016 to September 2016, students who had completed their research internship and handed in their assessment forms at the student service desk, were invited to participate in the qualitative part of this study. Written informed consent was obtained. Ethical approval for this study was obtained by the Ethical Review Board of the NVMO (Netherlands Association for Medical Education).

### Data collection

#### Quantitative data

Data (from September 2013 to September 2016) regarding the duration of the internships, location (the Netherlands or abroad), and grades for the research internship were retrieved from the student administration database.

Publications from students who performed their internship from September 2012 to September 2014 were searched in PubMed using the students’ and supervisors’ names. Papers were retrieved up to 2 years after completion of the internship, taking into consideration the delay in publication. All publications found were checked by hand, verifying the dates, the location, and the research subject. To be included in the analysis, papers needed to be published as peer-reviewed manuscripts in international scientific journals, describing the results of the research internships and (co-)authored by the student.

#### Qualitative data

We used an open-ended questionnaire to explore the motivation of students underlying their choice to do their research internship *before* or *after* their clinical clerkships. In this questionnaire students who had finished their research internship between February 2016 and September 2016 were asked to

- fill out the duration and location of the research internship
- give, retrospectively, the reasons which made them choose for performing their research internship *before* or *after* their clinical clerkships,
- indicate (if applicable) reasons why they would, in retrospect, make the same or another choice.

### Data analysis

#### Quantitative data

Descriptive statistics (means and percentages) were used for analysis. To test group differences, odd’s ratios were calculated and a t-test was performed where appropriate.

#### Qualitative data

We analysed the questionnaires of students who performed their research internship in the first or third year of their Masters’ programme separately. Reflexivity was a critical aspect in the analytical process. The research team consisted of researchers who have grounding and expertise in SDT (RAK, GC, AW) and researchers who have experience in the practice of education (IJvW, HEMD, RAK, GC). Two authors (IJvW and RAK) familiarized themselves with the data and continued by open coding of all reasons given on the open-ended questions. This initial open coding was done blinded for the groups and followed by independently creating categories by means of directed content analysis [19]. Directed content analysis is a method for coding qualitative data in which an existing theory (in this case SDT) is used for identifying the initial codes in the data and any codes that fall outside the theory are added as extra codes. The identified categories of reasons to perform the research internship *before* or *after* the clinical clerkships were categorised into different motivation types: intrinsic regulation, identified regulation, introjected regulation and external regulation for research, using SDT as a theoretical framework [18]. Results were compiled through discussion and any disagreements were resolved through consensus. Although the use of SDT as a theoretical framework for our analysis allows for a deeper understanding of students’ motivation for research, this focus may also cause us to leave other relevant aspects unnoticed. A frequency analysis was performed for the occurrence of all reasons.

## Results

### Comparison of research internship length, location, grades and publications

Data of a total of 797 research internships, completed between 2013 and 2016, were analysed for internship length, location and grades. Of these, 338 (42%) were performed *before* and 459 (58%) *after* clinical clerkships. We found that students who performed their research internship *before* their clinical clerkships more often chose for an extended research internship (OR = 3.16, 95% CI = 2.32–4.31) and for a research internship abroad (OR = 2.22, 95% CI = 1.46–3.36) compared to students who performed their research internship *after* the clinical clerkships (Table 1). Final grades for the research internships (assessing whether the student attained the learning objectives) did not differ (*p* = 0.51) between the two groups (Table 1). Moreover, internships performed *before* and *after* clinical clerkships showed similar numbers of publications. Papers were retrieved up to 2 years after completion of internships performed from 2012 to 2014. In total 551 students performed their research internships; 66 out of 242 students (27%) published a paper following a research internship *before* clinical clerkships and 87 out of 309 students (28%) published a paper following a research internship *after* clinical clerkships.

**Table 1:** Comparison of duration, location and grades of research internships

2013–2016 (*n* = 797)	*Before* clinical clerkships (*n* = 338)	*After* clinical clerkships (*n* = 459)	Odds Ratios [95% CI]
Choice for an extended internship, n (%)	155 (46)	97 (21)	OR = 3.16 [2.32–4.31]
Choice for an internship abroad, n (%)	63 (19)	43 (9)	OR = 2.22 [1.46–3.36]
Grades (1–10): mean (SD)	8.04 (0.8)	8.08 (0.8)	–

Regular research internship = 16 weeks, extended research internship = 24 weeks

Grades: 1–10 (1 = highly insufficient; 10 = excellent)

*SD* standard deviation, *OR* odds ratio, *CI* confidence interval

### Students’ reasons for their choices

**Fig. 2:**
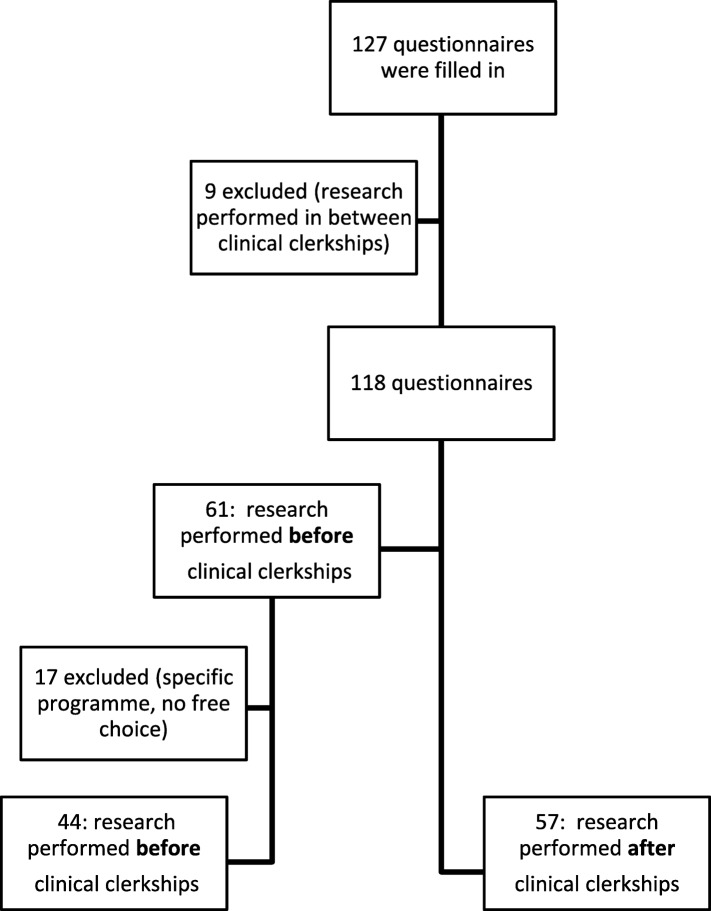
In- and exclusion of questionnaires of all participants

From February 2016 to September 2016, all students handing in their assessment forms of their internships, were asked to fill out a questionnaire. Of these 127 students, 61 students performed their research internship *before* and 57 *after* their clinical clerkships. Students who performed their internship in-between their clinical clerkships (a temporary option, *n* = 9) and students who were in a specific (honours) programme with a predefined time of their research internship (no free choice, *n* = 17) were excluded from further analysis (see Fig. 2).

This resulted in a total of 101 questionnaires which were analysed. Forty four percent (*n* = 44) of students chose to do their research internship *before* their clinical clerkships, whereas 56% (*n* = 57) chose to do it *after* their clinical clerkships. Also in this subset of students a significantly higher number of students performing their research internship *before* clinical clerkships chose for an extended research internship compared to the students doing their internships *after* clinical clerkships (42 and 19%, respectively). This is in accordance with analyses on all internships from 2013 to 2016 (see Table 1) and indicates that this is a representative subset of students in this respect.

Reasons to choose for either a research internship *before* or *after* clinical clerkships were collected, analysed and categorised according to the type of motivation. The result of this categorisation is shown in Table 2, including some examples of students’ quotes. The corresponding quantitative analysis is shown in Table 3. Quotes lacking any indication of the type of motivation or inconclusive statements, were excluded from further analysis (*n* = 6). Finally, three types of motivation for research could be extracted: intrinsic regulation for research, identified regulation for research and extrinsic regulation for research. No introjected regulation was seen.

**Table 2:** Illustration of student quotes and reasons, categorised in types of motivation for research

Type of motivation	Student’s reason for the choice made	Student quotes (some examples of quotes are given)	
		Research performed *before* clinical clerkships (*n* = 44)	Research performed *after* clinical clerkships (*n* = 57)
Intrinsic motivation for research	Students have a genuine interest in research	“I was motivated by an earlier research experience during the Bachelor” “I was already participating in research” “I wanted to continue my research project during clinical clerkships”	“I believed I would have more knowledge on research (and/or: interest in research) which could be beneficial for my research internship”
Identified regulation for research	Students find it personally important to gain experience in research or be good in research.	“I wanted to gain experience in research”	“I assumed that my clinical knowledge would be beneficial for the research internship” “Improve perspectives to obtain a position as a PhD-student”
Extrinsic regulation for research	Students are making this choice for an extrinsic goal not directly related to research	“I had a long wait / I had re-exams prior to the start of clinical rotations” “Easier to obtain a position as a resident for specialization” “I found a good opportunity to go abroad” “After the clinical rotations I would like to start directly with clinical work, not research” “I was not motivated (yet) to start the clinical rotations” “A nice opportunity came by”	“I wanted to choose a discipline corresponding to my future residency” “Personal external financial reasons” “Improve perspectives to obtain a position as a resident for specialization” “I was not motivated for research in year 1” “Wanted to start with clinical clerkships first” “This planning was in line with my personal schedule”

**Table 3:** Frequency of student quotes

Type of motivation for research	Frequency of quotes belonging to a certain type of motivation	
	Research internship performed *before* clinical clerkships, *n* = 44	Research internship performed *after* clinical clerkships, *n* = 57
Intrinsic motivation for research, n (%)	21 (26)	6 (6)
Identified regulation for research, n (%)	5 (6)	15 (16)
Extrinsic regulation for research, n (%)	56 (68)	74 (78)
Total number of reasons mentioned	82	95

Numbers indicate the frequency a quote/reason was given by students in the questionnaires. Students were allowed to mention more than one reason

The most frequently mentioned reason to opt for a research internship *before* clinical clerkships was to avoid waiting time (17 of 82; 21%). This is a purely pragmatic reason and can be considered extrinsic regulation for research. Similarly, the most frequently mentioned reason to opt for a research internship *after* clinical clerkships was that students wanted to choose the research internship within a discipline corresponding to their future residency (48 of 95; 50%), which can also be considered extrinsic regulation for research. Various other reasons given by students were based on extrinsic and identified regulations for research, such as the opportunity to go abroad or the advantage of a certain career choice (see Table 2).

Besides extrinsic and identified regulation, also reasons based on intrinsic motivation for research were seen. In the group of students who chose to perform their research internship *before* the clinical clerkship, students expressed their genuine interest in research or wanted to continue the research they had already participated in (21 of 82; 26%) during their Bachelor’s (which was extracurricular). In the other group of students, choosing to do their research internship *after* clinical clerkships, students believed they had more knowledge on (and/or interest in) research *after* the clinical clerkships (6 of 95; 6%). In research internships conducted *before* clinical clerkships choices were more often based on intrinsic motivation compared to internships *after* clinical rotations (26% versus 6%, Table 3).

In general, students were happy with the choice they had made. After completion of the internships, 86% (38/44) of students who performed their research internship *before* their clinical clerkships and 82% (47/57) of students who performed them *after* their clinical clerkships did not regret the choice they had made.

## Discussion

Research internships in a medical curriculum provide hands-on experience in research for medical students. This experience is pivotal for the preparation of students for participating in research and for critical evaluation of research outcomes as a practicing physician. In the current study we explored the timing of a research internship in a medical curriculum and found that academic grades and number of publications were comparable between students who performed their research internships *before* and *after* their clinical clerkships. According to these results we conclude that students seem to attain the learning goals of the research training equally well in both scenarios. Hence a research internship might be positioned in the beginning as well as at the end of a medical curriculum.

Interestingly, accompanying choices made by students on duration and location of the internship and the motivation of students do differ. Students who perform research at the start of their medical school significantly more often chose to do an internship abroad and an extended internship (24 weeks instead of 16 weeks). This willingness to spend more time on the research internship, might be indicative of a higher (intrinsic) motivation to perform research. Indeed, these students more often expressed reasons based on intrinsic motivation for research to choose for a research internship performed *before* clinical clerkships (26% compared to 6% in the group of students who chose to perform their research internship *after* clinical clerkships). Hence, positioning of the research internship at the beginning of the medical school curriculum might be advantageous for students who are motivated to do research and who are already involved in research. These students can opt for continuing their research during their following medical school years. Studies of O’Sullivan et al., Amgad et al. and Peacock and Grande show the advantages of stimulating students to perform research as early as possible in their curriculum, supporting the position of the research experience early in the curriculum [7, 13, 14].

Whether this timing is the best option for all students is questionable. In the group of students who performed their research internship *after* the clinical rotations, we observed that other reasons seem to be important; half of the students (50%) want to perform the research in line with their future specialization. As they do not know their preferred specialization at the start of their Master’s curriculum, they prefer to postpone the research internship. This reason is related to their career choice and based on extrinsic regulation for research. However, it is important to realize that these reasons reflect an identified regulation type of motivation for the medical study as a whole; students are motivated to make the best choice for their medial career and become a good doctor. Therefore, these students benefit from their personal choice to perform research *after* their clinical clerkships. As a consequence, different groups of student might benefit from either of both positions of the research experience in the curriculum.

In our student population we did not observe more (intrinsic) motivation for research following cumulative clinical experience as shown by Rosenkranz et al. [15]. This might be explained by differences in prior training and education on research in the curriculum. However, our study supports their conclusions that motivation for research might be enhanced by increasing students’ sense of autonomy (e.g. options to choose), competence (confidence and a sense of mastery) and relatedness to research (relevance and importance of research).

We acknowledge the limitations of our study. The current study is a single-center retrospective study. As many variables on the research experience might differ in other schools (e.g. the duration, learning goals, mentoring, whether it is mandatory or not), the findings might not be applicable to all medical schools. Using publications alongside grades, to analyse whether students attain the learning goals of the research training, is another limitation. Negative research results may not be published, possibly leading to confounding. Collecting data on abstracts and conference presentations might be a valuable addition. In this study we investigated views of students in a cross-sectional manner, directly after finishing the internship. It would be interesting to investigate if the students who were confident of their choice to perform the research internship *before* clinical rotations, would think differently after graduation or not. Finally, by categorizing and subsequently analysing the type of motivation of students, we might have neglected confounders which might have played a significant role in the choice they made. External parameters like e.g. the financial situation might differ between both groups and play a role in the choices made. Focus group discussions with students might contribute to obtain a broader impression of all factors involved in their choices made.

A strong aspect of the current study is the combination of the qualitative and quantitative approach, giving unique insights into the consequences of different timings of research internships in a medical school curriculum. Moreover, the very design of our curriculum allowing students a choice, has enabled us comparison of the two different positions of the research internship.

In future studies it will be interesting to analyse the outcomes of research internships as a function of both positioning (*before* or *after* clinical clerkships) and motivation for research simultaneously in a 2 × 2 format. Our current experimental design did not permit this. In addition, future work might focus on the influence of the positioning of the research internship in the curriculum on the students’ career choices after graduation. This could reveal long term effects of the choices made by the students.

Based on our data, a minority of the students seem to be intrinsically motivated to perform research. Many students do not seem to be motivated to learn about research even if they are intrinsically motivated for the medical study; there might be a discrepancy between interest in clinical practice and interest in research. Many students choose the timing of their research internship in line with their chances to obtain a place for specialization. A lack of appreciation of research among medical students has been reported before [7, 15, 20], indeed Amgad et al. [7] confirmed, analysing seven relevant studies, that the main motive for students to participate in research is career progression.

Therefore it is important to think about effective ways to motivate students for research. In general, offering students different choices (on timing of the internship, location and research project) might enhance their sense of autonomy and thereby improve their motivation and learning outcomes [21, 22]. Moreover, motivation for research might specifically be enhanced by influencing students’ perception of research [15]. Right at the beginning of the medical studies, role models and mentors should stress the indispensable role of research in a medical practice, so that students come to value research and consider it personally important.

## Conclusion

As students have different types of motivation, interests and ambitions, the best way to position the research internship in a medical curriculum might be to accommodate these internships not only *after*, but also *before* the clinical clerkships. We recommend some flexibility and choice in scheduling the research internship, which will facilitate students who show an early interest in and motivation for research, and facilitate students who want to postpone their research internship until they made their choice for a future specialization.
